# SMOC2, an intestinal stem cell marker, is an independent prognostic marker associated with better survival in colorectal cancers

**DOI:** 10.1038/s41598-020-71643-1

**Published:** 2020-09-03

**Authors:** Bo Gun Jang, Hye Sung Kim, Jeong Mo Bae, Woo Ho Kim, Heung Up Kim, Gyeong Hoon Kang

**Affiliations:** 1 Department of Pathology, Jeju National University School of Medicine, Jeju, Korea; 2 Department of Internal Medicine, Jeju National University School of Medicine, Aran 13gil 15, Jeju, 690-767 Korea; 3 Department of Pathology, Seoul National University College of Medicine, 103 Daehak-ro, Jongno-gu, Seoul, 110-799 Korea; 4 Laboratory of Epigenetics, Cancer Research Institute, Seoul National University College of Medicine, Seoul, Korea

**Keywords:** Colorectal cancer, Prognostic markers

## Abstract

We aimed to investigate the expression profile of SPARC-related modular calcium-binding protein 2 (SMOC2) during colorectal cancer (CRC) progression and assess its prognostic impact in CRC patients. In our study, we showed that *SMOC2* transcript level was higher in CRC samples than in normal mucosa (*P* = 0.017); this level was not associated with candidate cancer stem cell markers (*CD44*, *CD166*, *CD133*, and *CD24*) or intestinal stem cell markers (*LGR5*,* ASCL2*, and *EPHB2*) except for *OLFM4* (*P* = 0.04). Immunohistochemical analysis showed that SMOC2-positive cells were confined to the crypt bases in the normal intestinal mucosa, hyperplastic polyps, and sessile serrated adenomas, whereas traditional serrated adenomas and conventional adenomas exhibited focal or diffuse distribution patterns. In total, 28% of 591 CRCs were positive for SMOC2, but SMOC2 positivity had negative correlations with lymphatic invasion (*P* = 0.002), venous invasion (*P* = 0.002), and tumor stage (*P* < 0.001). However, a positive association with nuclear β-catenin expression was seen. Furthermore, while upregulated SMOC2 expression was maintained during the adenoma-carcinoma transition, it decreased in cancer cells at the invasive front but did not decline further during lymph node metastasis. SMOC2 positivity showed no correlations with molecular abnormalities, including microsatellite instability, CpG island methylator phenotype, and mutations of *KRAS* and *BRAF*. In addition, we showed comprehensively that SMOC2 positivity is an independent prognostic marker for better clinical outcomes in a large cohort of CRC patients (*P* = 0.006). In vitro studies also demonstrated that induced SMOC2 expression in DLD1 cells exerts a suppressive role in tumor growth as well as in migration, colony, and sphere formation abilities. Taken together, our results suggest SMOC2 as a candidate tumor suppressor in CRC progression.

## Introduction

SPARC-related modular calcium-binding protein 2 (SMOC2) is an extracellular glycoprotein, comprised of thyroglobulin type-I domains, EF-hand calcium-binding domains, and a follistatin-like domain, and is widely expressed in many tissues. SMOC2 is involved in a variety of cellular processes including cell cycle^1^, cell attachment and migration^2^, angiogenesis^3^, fibrosis^4,5^, and tissue calcification^6^. Moreover, SMOC2 has been implicated in various human pathologies. For instance, *SMOC2* polymorphism has been associated with autoimmune diseases, such as generalized vitiligo and autoimmune thyroid disease^7,8^. Furthermore, *SMOC2* (G>A) polymorphism was reported to be significantly associated with primary glaucoma^9^. Additionally, *SMOC2* mutations have been identified in several dental disorders, including a severe developmental dental defect^10^ and oligodontia and microdontia^11^. Lastly, SMOC2 expression and its functional significance has been explored in many types of malignancies, such as colon^12^, breast^1^, liver^13^, lung^14^ and endometrial^15^ cancers.

Remarkably, SMOC2 expression was identified to be enriched in LGR5-positve stem cells at the bottom of intestinal crypts, and SMOC2-positive cells have been shown to generate the entire crypt cells by lineage tracing^16^. This finding proved SMOC2 is an intestinal stem cell (ISC) marker, implying that SMOC2 may play an important role in colon cancer development. Since then, however, there have been only a few studies that investigate the significance of SMOC2 expression in colorectal cancer (CRC) progression; Shvab et al*.* showed that SMOC2 elevation is necessary for the increase in cell motility, proliferation and liver metastasis in colon cancers^12^. Whereas Kim et al*.* demonstrated that SMOC2 expression substantially increases during colitis-associated carcinogenesis with other ISC markers, such as LGR5 and ASCL2^17^. There is no study that has investigated the prognostic impact of SMOC2 in CRC patients. Therefore, we aimed to examine the expression profile of SMOC2 in various precancerous colorectal lesions, as well as in a large cohort of CRCs to evaluate its prognostic significance. In particular, we analyzed alterations in the expression of SMOC2 during CRC progression: adenoma-carcinoma transition, muscle layer invasion, and lymph node metastasis. Furthermore, the functional significance of SMOC2 on cancer growth and migration were also explored using CRC cell lines.

## Materials and methods

### Subjects

A large number of CRCs samples (n = 734) were collected from the patients who underwent curative or palliative surgical resection at Seoul National University Hospital (SNUH) (Seoul, Korea) between 2004 and 2008. Patients were excluded from the study if they met any of the following criteria: receiving neoadjuvant chemotherapy, familial polyposis, ulcerative colitis, or Crohn's disease. In total, 396 patients were treated with one of the following post-operative chemotherapy regimens: FL (5-fluorouracil and leucovorin), FOLFOX (oxaliplatin, leucovorin, and 5-fluorouracil), FOLFIRI (irinotecan, leucovorin, and 5-fluorouracil), XELOX (oxaliplatin and capecitabine), XELODA (Capecitabine), and others. Among them, 36 patients received concurrent radiation therapy. Clinicopathological data including patient age, gender, tumor location, tumor size, histological type, presence of lympho-vascular and perineural invasion, American Joint Committee on Cancer/International Union against Cancer (AJCC/UICC) cancer stage (7th edition), time of tumor recurrence, time of death and follow-up time were obtained by thoroughly reviewing the clinical and pathological reports. Median follow-up time was 57 months (mean ± SD: 50 ± 23 months). Two clinical end points were used. Recurrence-free survival was the time from surgery to first evidence of tumor recurrence; overall survival was the time from surgery to death from any cause. Tumor differentiation was evaluated according to a three-tier grading system as described in the World Health Organization (WHO) classification of tumors of the digestive system. Presence of tumor budding was defined when a single cancer cell or a small cluster of cancer cells is observed at the invasive margin. The histopathologic features of CRCs were evaluated by two gastrointestinal pathologists (J.M.B and G.H.K). Also, 206 CRC samples were collected from the patients who had surgical resection at Jeju National University Hospital (JNUH) (Jeju, Korea); 24 cases are CRCs arising from preexisting adenomas and 182 cases are conventional CRCs, clinicopathological characteristics of which have been described in the previous study. Additionally, 28 paired-fresh CRC tissues and corresponding normal tissues were provided by the Jeju National University Hospital Biobank, a member of the National Biobank of Korea and informed consent was obtained from the subjects. This study was approved by the Institutional Review Board of SNUH (C-1502-029-647) and JNUH (2016-11-012), respectively and all procedures were in accordance with the Helsingki Declaration of 1964 and later versions.

### DNA extraction

Genomic DNA was isolated from the formalin-fixed and paraffin-embedded (FFPE) as described previously^18^. Briefly, FFPE tissues of 734 CRCs were retrieved from SNUH pathology archives. A representative tumor area (tumor cells > 70% of marked area) was microdissected with a surgical blade from 10 μm-thick tissues. The tumor tissues were digested in lysis buffer (100 mM Tris–HCl, 10 mM EDTA, 1 mg/ml proteinase K, and 0.05 mg/ml tRNA) at 55 °C for 48 h, followed by a 95 °C incubation for 10 min to inactivate proteinase K. The extracted genomic DNA was stored at − 20 °C to be used for subsequent molecular studies such as *KRAS*/*BRAF* mutations, MSI, and CIMP analyses.

### Microsatellite instability (MSI) analysis

All 734 CRC samples were subjected to MSI analysis with the fluorescent multiplex PCR method using five NCI recommended microsatellite markers (BAT25, BAT-26, D5S346, D17S250, and D2S123)^19^. The MSI status of each case was classified into three categories: MSI-high (two or more unstable markers in the five markers), MSI-low (one unstable marker in the five markers) and microsatellite stable (no unstable marker in the five markers).

### DNA methylation analysis

DNA analysis to determine CpG island methylation phenotype (CIMP) status was carried out as described previously^20^. In brief, Sodium bisulphite modification of genomic DNA was performed for all CRC tissues. The quantitative measurement of the promoter CpG island methylation of eight CIMP genes (*MLH1*, *NEUROG1*, *CRABP1*, *CACNA1G*, *CDKN2A*, *IGF2*, *SOCS1*, and *RUNX3*) was performed with the methylation specific real-time PCR method (MethyLight assay). A CIMP-high CRC was defined as having five or more hypermethylated markers, a CIMP-low CRC as having one to four hypermethylated markers, and a CIMP-negative CRC as having no hypermethylated marker. A hypermethylated CpG island locus was defined when the percentage of the methylated reference (PMR) value was over 4. The MethyLight assay for each CIMP marker was repeated three times, independently. The final determination of the promoter hypermethylation was made when a PMR value > 4 was observed in at least two of three experiments.

### KRAS/BRAF mutation analysis

Mutation analysis for *KRAS*/*BRAF* gene was performed as previously described^20^. Mutations of *KRAS* codons 12 and 13 and *BRAF* codon 600 were assessed using PCR-restriction fragment length polymorphism and direct sequencing techniques. Among 788 CRCs examined in this study, due to an insufficient DNA amount, 39 and 81 samples were excluded from the *KRAS* and *BRAF* mutation analyses, respectively.

### Tissue microarray (TMA) construction

In total, 20 TMAs containing 734 CRCs from SNUH were constructed as previously described^20^. Through histologic examination, one representative tumor portion comprising more than 70% of cell population was marked at the tumor center in each case. For CRCs from SNUH, core tissue biopsies of 2 mm in diameter were obtained from individual FFPE colorectal cancers (donor blocks) and arranged in a new recipient paraffin block (tissue array block) using a trephine apparatus (SuperBioChips Laboratories, Seoul, Korea). For CRCs from JNUH, 2 TMAs containing 24 pairs of adenoma and carcinoma portions and 15 TMAs containing 182 cases of CRCs were constructed with cores of 4 mm in diameter. For ulcero-fungating cancers, both superficial and invasive areas were included, and if present, metastatic cancers in the lymph node also were included.

### Immunohistochemistry and interpretation

Immunohistochemistry for SMOC2 and β-catenin and were performed on 4-μm TMA sections using a BOND-MAX automated immunostainer and a Bond Polymer Refine Detection kit (Leica Microsystems, Wetzlar, Germany) according to the manufacturer’s guidelines. The primary antibodies used were anti-SMOC2 (OriGene, Rockville, MD; 1:30, catalog number: TA351730) and anti-β-catenin (Novocastra Laboratories, Newcastle, UK; 17C2; 1:800). The expression of SMOC2 was determined by evaluating the whole field of each tumor core (4 mm in diameter). The intensity and percentage of tumor cells expressing SMOC2 in the cytoplasm were examined. Histoscores (H-scores) were measured by multiplying the intensity score (0 = negative; 1 = weak; 2 = moderate; 3 = strong) and percentage of positive tumor cells (range = 0–100), ranging from 0 to 300^21^. For statistical analyses, we set a cutoff of 40 on the basis of the distribution of H-scores (mean value: 32.5). CRCs with H-score < 40 were classified as negative, while cases with H-score > 40 were classified as positive. Nuclear β-catenin staining was defined as positive when more than 10% of the cancer nuclei were stained for β-catenin. Immunohistochemical analysis was performed blinded to all other data.

### RNA extraction and quantitative real-time PCR

RNA extraction and real-time PCR was performed as previously described^22^. In brief, Total RNA was isolated from 28 paired fresh-frozen CRCs and matched non-cancerous tissues with TRIZOL reagent (Invitrogen, Carlsbad, CA, catalog number: 15596018). RNA (1 to 2 μg) was subjected to reverse-transcription with oligo-dT primers and the GoScript reverse transcription system (Promega, Madison, WI, catalog number: A5004) to generate complementary DNA (cDNA). Subsequently, cDNA was used to perform real-time PCR with Premix Taq Hot Start Version (Takara Bio, Shiga, Japan, catalog number: R028A). The cycling condition was as follows: initial denaturation for 30 s at 95 °C, followed by 40 cycles of 95 °C for 1 s and 60 °C for 5 s in a StepOne Plus real-time PCR system (Applied Biosystems, USA). The TaqMan gene expression assays were used as follows: Hs00405777_m1 (SMOC2), Hs00173664_m1 (LGR5), Hs00270888_s1 (ASCL2), Hs00197437 (OLFM4), Hs00362096-m1 (EPHB2), Hs01075864_m1 (CD44), Hs00233455_m1 (CD166), Hs01009250_m1 (PROM1/CD133), and Hs0275899_g1 (GAPDH). GAPDH served as the endogenous control.

### Colon cancer cell lines

Twelve CRC cell lines (DLD1, HT29, SW620, HCT116, LoVo, KM12C, KM12SM, KM12L4, HCT116, LS174T, SW1116, and Colo205) were purchased from the Korean Cell Line Bank (https://cellbank.snu.ac.kr, Seoul, Korea). Cells were cultured in RPMI1640, MEM, DMEM, or L15 medium (Welgene, Daegu, Korea) at 37 °C in a humidified incubator with 5% CO2 according to the guidelines. All culture media contained 10% fetal bovine serum (FBS) (Gibco, Carlsbad, CA) and 1% penicillin/streptomycin (Gibco).

### Antibodies and reagents

Anti-SMOC2 was purchased from OriGene (catalog number: TA351730). Anti-β-actin antibody was purchased from Abcam (Cambridge, UK, catalog number: ab6276). The anti-caspase-3 (catalog number: #9662), anti-cleaved caspase-3 (catalog number: #9661), anti-beta-catenin (catalog number: #9562), anti-ERK (catalog number: #4695), anti-AKT (catalog number: #4691), anti-phospho-ERK (catalog number: #4370), and anti-phospho-AKT (catalog number: #4060), anti-cleaved PARP (catalog number: #5625), anti-BAX (catalog number: #5023), and anti-BIM (catalog number: #2933) antibodies were purchased from Cell Signaling Technology (Danvers, MA, USA). Anti-LaminB1 (catalog number: sc-365214), anti-mouse IgG-HRP (catalog number: sc-2031) and anti-rabbit IgG-HRP (catalog number: sc-2030) antibodies were purchased from Santa Cruz Biotechnology (Santa Cruz, CA, USA). Geneticin was purchased from Sigma Aldrich (St Louis, MO, USA). Recombinant human SMOC2 protein was purchased from R&D systems (Minneapolis, MN, USA, catalog number: 5140-SM-050).

### Western blot analysis

Cellular proteins were prepared from the cell pellets using lysis buffer (iNtRON Biotechnology, Seongnam, Korea). To obtain nuclear and cytoplasmic fractions, a NE-PER nuclear and cytoplasmic extraction kit (Pierce, Rockford, IL, USA) was used according to the manufacturer’s instructions. The protein amount was measured using BCA protein assay kits (Pierce, Rockford, IL, USA). Lysates were separated on 10% SDS–polyacrylamide gel and electrophoretically transferred to PVDF membrane (Millipore Corporation, Bedford, MA), followed by blocking with 5% non-fat dry milk in PBS-Tween-20 (0.1%, vol/vol) for an hour. The membrane was incubated with a primary antibody at 4 °C for overnight. After being washed with TBS containing 0.1% Tween-20, the membrane was incubated for 1 h at room temperature with a secondary antibody. The target proteins were detected using the chemiluminescent reagents and visualized in Alliance-Mini.HD9 chemiluminescence documentation system (UVItec Cambridge, UK).

### Transfection and stable cell line generation

Plasmid DNA with full-length SMOC2 cDNA (pCMV6-SMOC2) was purchased from Origene. Cells were seeded at 1 × 10^6^ cells/well in a 6-well plate and transfected with 3 μg of pCMV6-SMOC2 (or control vector; pCMV6) using the Neon transfection system (Invitrogen, Carlsbad, CA, USA). One day after DNA transfection, cells were subjected to real-time PCR, western blot, growth and migration assays. All experiments were performed at least 2–3 times, independently. To generate stable cell lines expressing SMOC2, DLD1 cells were transfected with control or SMOC2-containing plasmid vector. One day after transfection, cells were treated with the 800 μg/ml geneticin for two to three weeks until the survival clones proliferate. Two control vector- and three SMOC2-expressing DLD1 cell lines were established and SMOC2 expression was confirmed by western blot.

### Caspase-3 activity assay

Caspase-3 enzymatic activity was measured using a Caspase-Glo 3 Assay Kit (Promega, Madison, WI, USA, catalog number: G8091). After transfection with control or SMOC2-containing vector, DLD1 cells (1 × 10^4^ cells/mL) were seeded and cultured in 96-well plates (100 μL/well) for 24 h in triplicate. Caspase-Glo Reagent (100 μL/well) was added to each well and the cells were incubated in the dark at room temperature for 3 h on a shaker. The luminescence in each well was measured using GloMax Navigator System (Promega).

### Proliferation assay, colony formation assay, and sphere formation assay

Cells were harvested 24 h after transfection and seeded at 5 × 10^3^ cells/well on a 96-well plate and incubated at 37 °C. At indicated time points, cell growth was evaluated by adding 10 μl of Cell Counting Kit-8 reagent (Dojindo, Kumamoto, Japan) into each well and incubating for an hour. Absorbance was measured at 450 nm using a spectrophotometer (Thermo Labsystems, Rockford, IL, USA). For a colony formation assay, 5 × 10^3^ to 1 × 10^4^ cells were counted using LUNA-II (Logos Biosystems, Gyeonggi-do, Korea) and plated in a 60-mm culture dish and incubated for 2 to 3 weeks until distinguishable tumor colonies appeared. Colonies were fixed with 70% methanol solution and stained with a 0.01–0.1% crystal violet solution. For a sphere formation assay, cells were plated at a density of 100 cells in a Cultrex 3D Basement Membrane Matrix (Trevigen, Gaithersburg, MD, USA) and cultured for 4 weeks and medium was changed every week.

### Wound healing assay and migration assay

For a wound healing assay, cells were transfected with the control or SMOC2 plasmid DNA and were cultured in a SPL Scar Block (SPL Life Sciences, Seongnam, Korea). Scar Block is composed of 500 μm-thick walls to generate cell free gaps. When cells are confluent, block was removed from the plate and culture medium was added. Cellular migration was monitored and photographed at 0 and 48 h. As a migration assay, transfected cells with control or SMOC2 plasmid DNA were seeded at upper well of 24-well culture plates with transwell inserts (pore size: 8 μm) (BD Bioscience, San Diego, CA, USA). The upper well of the transwell contained 2 × 10^5^ cells in 300 μL serum-free RPMI medium, whereas the lower well contained 500 μL RPMI with 10% FBS. After 24 h of incubation, non-migrated cells that remain on the top of transwell insert were removed with a cotton swab and cells that migrate through the pores and reach the bottom side were fixed with methanol for 10 min and counted after staining with crystal violet for 1 h.

### cDNA microarray analysis

The array process was executed by Macrogen Inc. (Seoul, Korea) as previously described^23^. Affymetrix Human Gene ST 2.0 arrays were used for the cDNA microarray analysis The Affymetrix Whole transcript Expression array process was executed according to the manufacturer's protocol (GeneChip Whole Transcript PLUS reagent Kit). cDNA was synthesized using the GeneChip WT (Whole Transcript) Amplification kit as described by the manufacturer. The sense cDNA was then fragmented and biotin-labeled with TdT (terminal deoxynucleotidyl transferase) using the GeneChip WT Terminal labeling kit. Approximately 5.5 μg of labeled DNA target was hybridized to the Affymetrix GeneChip Array at 45 °C for 16 h. Hybridized arrays were washed and stained on a GeneChip Fluidics Station 450 and scanned on a GCS3000 Scanner (Affymetrix) The data were summarized and normalized with robust multi-average (RMA) method implemented in Affymetrix Power Tools (APT). Statistical significance of the expression data was determined using LPE test and fold change in which the null hypothesis was that no difference exists among groups. False discovery rate (FDR) was controlled by adjusting p value using Benjamini–Hochberg algorithm. For a DEG set, Hierarchical cluster analysis was performed using complete linkage and Euclidean distance as a measure of similarity. All data analysis and visualization of differentially expressed genes was conducted using R 3.3.2 (www.r-project.org).

### Statistical analysis

Statistical analyses were performed using the SPPSS statistical software version 18.0 (SPSS, Chicago, IL) and Prism version 5.0 (GraphPad Software, San Diego, CA). The correlations between *SMOC2* and stem cell-related markers were evaluated by the Spearman correlation test. Between-group comparisons of the real-time PCR data or H-scores of SMOC2 were performed using Student’s t-test. H-scores of SMOC2 between benign colorectal lesions were compared using Tukey’s Multiple Comparison Test. Survival curves were estimated using Kaplan–Meier method and log-rank test was used to compare groups. Using Cox proportional hazards model, univariate and multivariate analyses were carried out to identify independent prognostic factors. A *P*-value < 0.05 was considered statistically significant.

## Results

### SMOC2 expression in colorectal cancers and its correlation with stem cell-related markers

**Figure 1:**
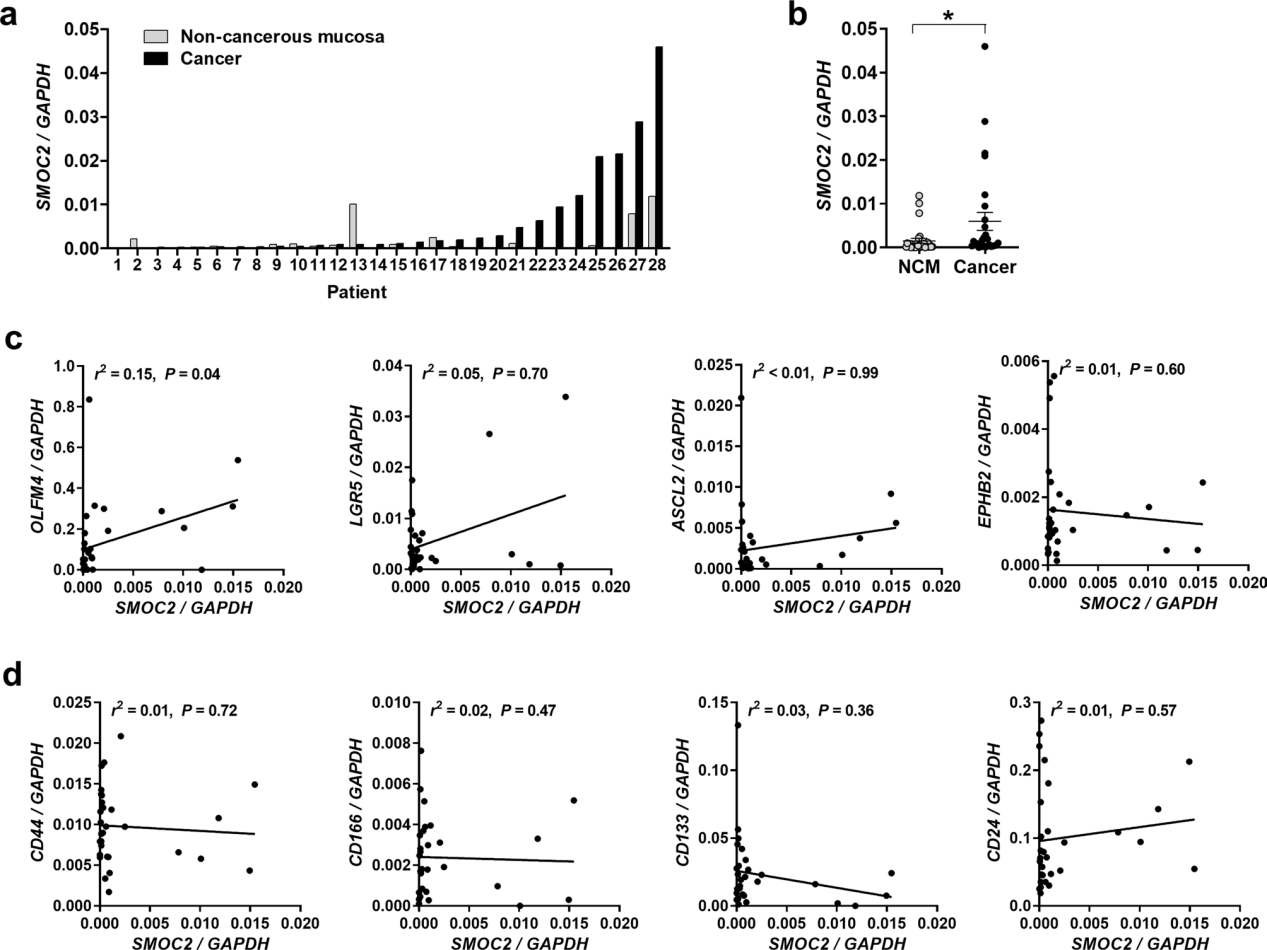
SMOC2 mRNA expression in colorectal cancers (CRCs) and its correlation with intestinal stem cell (ISC) and cancer stem cell (CSC) markers. (**a**, **b**) Real-time PCR analysis measured the expression of *SMOC2* and ISC (*OLFM4, LGR5, ASCL2*, and *EPHB2*) and CSC markers (*CD44, CD166, CD133*, and *CD24*) from 28 pairs of fresh-frozen CRCs and matched non-cancerous mucosa (NCM). *SMOC2* mRNA expression was significantly higher in CRCs than in NCM. (**c**, **d**) Scatter plots showing the correlations between *SMOC2* and ISC or CSC markers expression. Data are presented as the mean ± SD. *P < 0.05.

To determine the expression of SMOC2 and stem cell-related markers in CRCs, real-time PCR was performed on a series of 28 pairs of fresh CRC samples as well as on matched normal colonic tissues. Compared to normal mucosa (mean ± SD: 0.0015 ± 0.0031), *SMOC2* mRNA levels were significantly higher in in 43% of CRC samples examined (12 out of 28 cases) (Fig. 1a). The mean *SMOC2* expression level was higher in CRCs (mean ± SD: 0.0060 ± 0.0108) than in matched normal tissues (*P* = 0.017) (Fig. 1b). As *SMOC2* is enriched in the stem cells of the intestinal crypts, we examined whether there is any correlation between *SMOC2* and other ISC markers, including *LGR5*, *ASCL2*, *EPHB2*, and *OLFM4*. We found that only *OLFM4* demonstrated a positive association with *SMOC2* (*r*^2^ = 0.15, *P* = 0.04) (Fig. 1c), suggesting that the close relationship between ISC signature genes observed in the normal stem cell niche is disrupted during cancer development. In addition, since a stem cell marker in the normal crypts is considered as a promising cancer stem cell (CSC) marker, we also investigated the association of *SMOC2* with candidate CSC markers in human CRCs, such as *CD44*, *CD16*6, *CD133*, and *CD24*. However, none of them showed significant correlations with *SMOC2* expression (Fig. 1d).

### SMOC2 expression in normal mucosa and precancerous lesions

**Figure 2:**
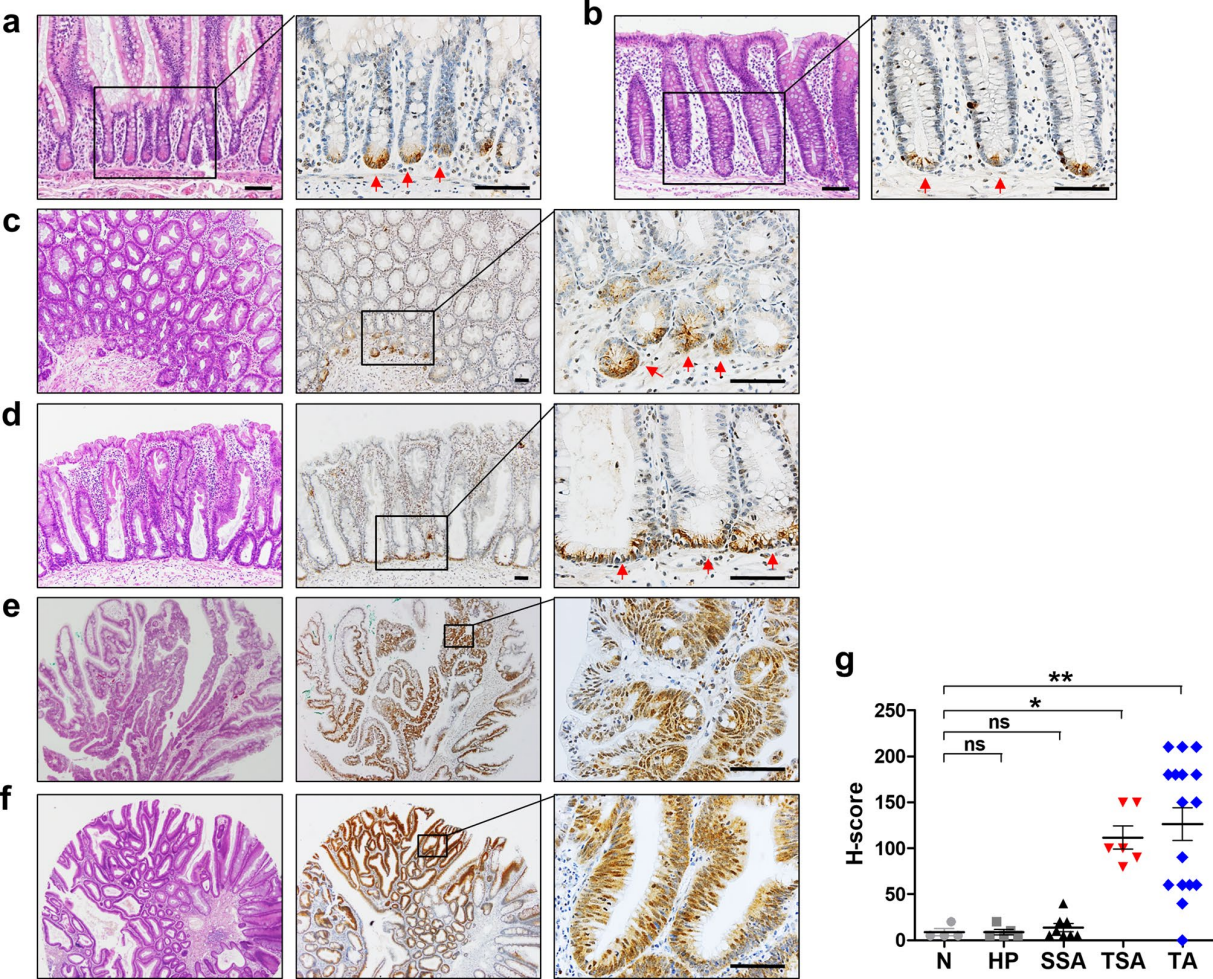
Immunohistochemical analysis for SMOC2 expression in normal and various colorectal precancerous lesions. SMOC2 expression was observed at the bottom of crypts in the small (**a**) and large intestinal mucosa (**b**). SMOC2 expression was restricted at the basal areas of hyperplastic polyps (HPs) (**c**) and sessile serrated adenomas (SSAs) (**d**). (**e**) Traditional serrated adenomas (TSAs) tended to exhibit strong SMOC2 expression in ectopic crypt foci. (**f**) Conventional tubular adenomas (TAs) showed strong SMOC2 expression in either a focal or diffuse manner. (**g**) Histoscores (H-scores) for normal mucosa (N, n = 4), HPs (n = 5), SSAs (n = 8), TSAs (n = 6), TAs (n = 17). Black boxed areas are shown in higher magnification in the right next panels. Scale bars: 50 μm.

We investigated the expression profile of SMOC2 in normal intestinal mucosa and various precancerous lesions. Immunohistochemical analysis on normal small (Fig. 2a) and large intestines (Fig. 2b) clearly showed a specific marking of SMOC2-positive cells at the crypt bases. This finding is in agreement with other previous reports demonstrating the presence of SMOC-expressing cells at the bottom of crypts in mice and humans^12,16^. Interestingly, in hyperplastic polyps (HPs) (Fig. 2c) and sessile serrated adenomas (SSAs) (Fig. 2d), SMOC2 expression remained at the bottom of the crypts with weak to moderate stain intensity. On the other hand, traditional serrated adenomas (TSAs) and tubular adenomas (TAs) exhibited focal or diffuse patterns of SMOC2 expression with moderate to strong stain intensity (Fig. 2e,f). Overall, SMOC2 expression was substantially higher in TSAs and TAs compared to that in HPs and SSAs (Fig. 2g).

### Altered expression of SMOC2 during CRC progression

**Figure 3:**
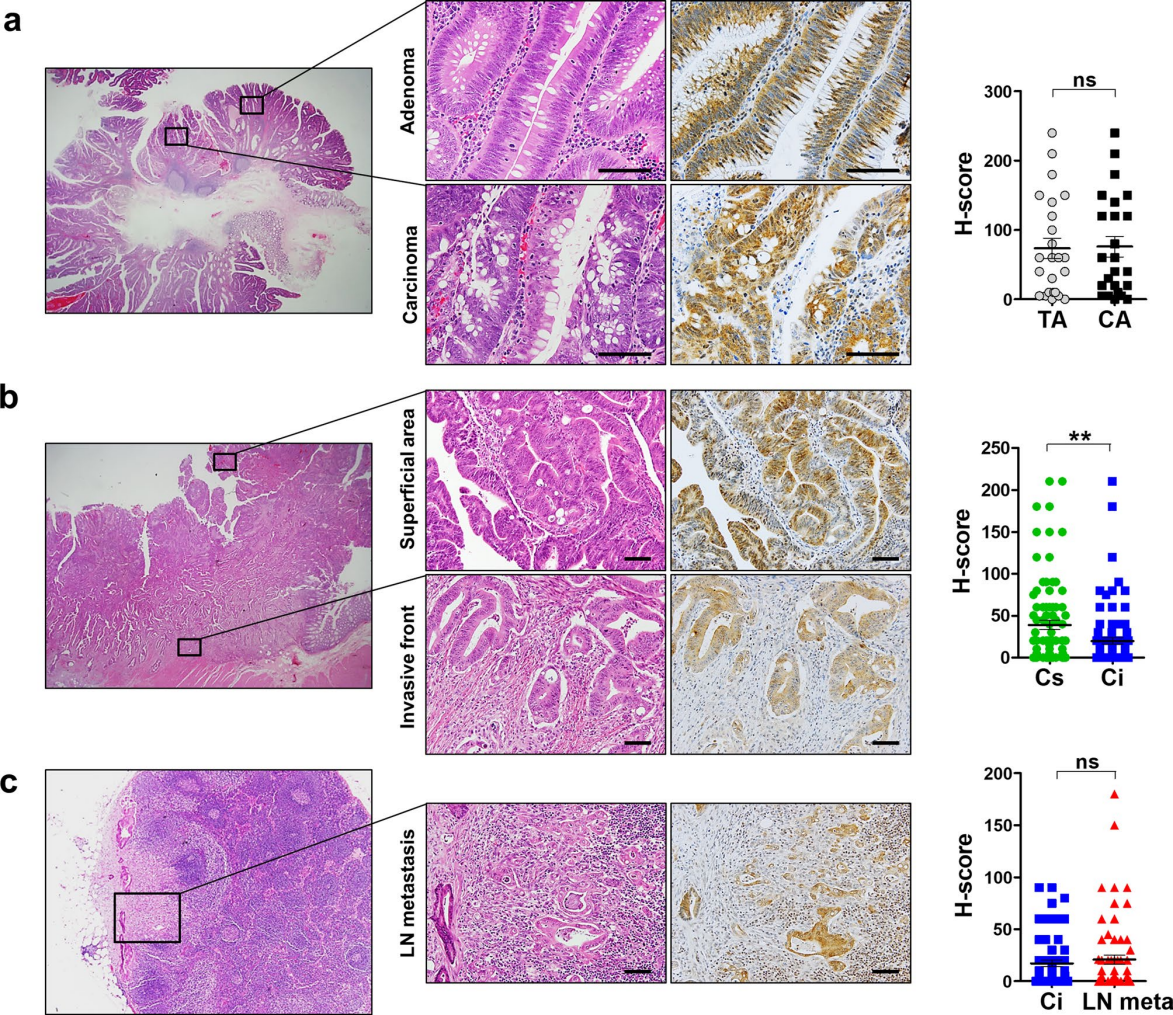
SMOC2 expression profile during colorectal cancer progression. (**a**) Immunohistochemical analysis for SMOC2 was performed on tissue microarrays containing colorectal adenomas and cancers. There was no significant difference in SMOC2 expression between adenoma and carcinoma portions in CRCs arising from pre-existing adenomas (n = 23). SMOC2 expression significantly declined in the invasive fronts than in superficial areas in ulcero-fungating cancers (n = 24) (**b**); however, no difference was observed between invasive fronts and metastatic cancer cells in the lymph node (**c**). Black boxed areas are shown in higher magnification in the middle panels. **P < 0.01, ***P < 0.001. Scale bars: 50 μm.

To determine differential SMOC2 expression over adenoma-carcinoma progression, we collected 24 cases of CRCs that co-exist with pre-existing adenomas. Comparing the H-scores of SMOC2 between adenoma and carcinoma portions, no significant difference was observed (Fig. 3a). Next, we assessed a number of ulcero-fungating type CRCs (n = 23); for each cancer we compared H-scores of SMOC2 from two different spots: superficial (or fungating) area, and invasive front. Notably, SMOC2 expression was significantly reduced in the cancer cells at invasive fronts (n = 89, Fig. 3b). Moreover, when comparing SMOC2 expression between primary cancer cells at the invasive fronts and metastatic cancer cells in the lymph node, no significant difference was observed (n = 65, Fig. 3c). These results suggest that SMOC2 down-regulation mostly occurs at the invasive fronts rather than during adenoma-carcinoma transition or lymph node metastasis.

### Clinicopathological and prognostic significance of SMOC2 expression in CRCs

**Figure 4:**
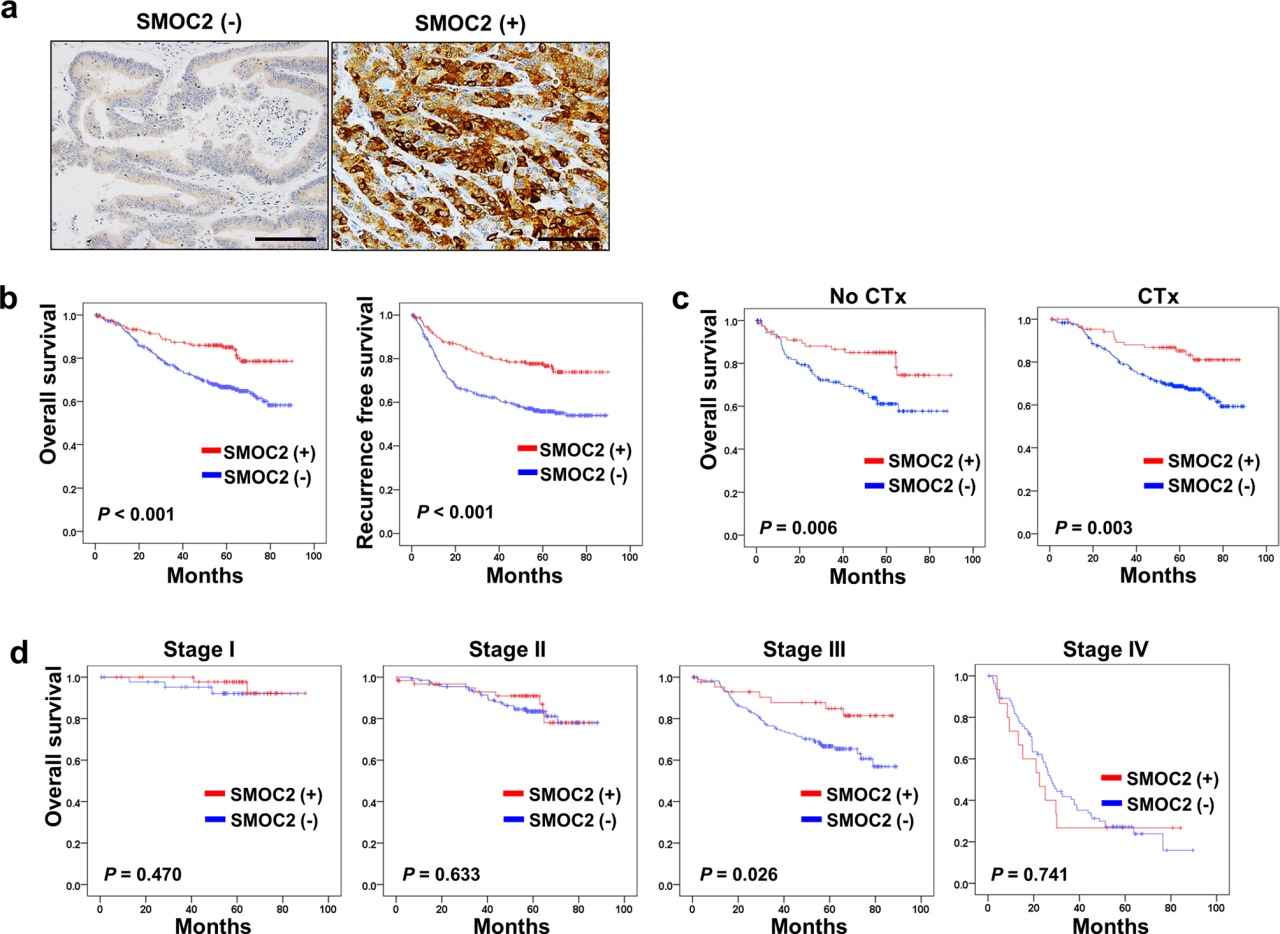
Overall and recurrence-free survival of colorectal cancer (CRC) patients with SMOC2 expression. (**a**) Immunohistochemistry for SMOC2 was performed in a large cohort of CRCs (n = 591). Representative cases of SMOC2-negative and SMOC2-positive colorectal cancers. (**b**) Kaplan–Meier analysis demonstrated that SMOC2 positivity showed better overall (P < 0.001), and recurrence-free (P < 0.001), survival rates. (**c**) SMOC2-positive patients showed improved clinical outcomes regardless of adjuvant chemotherapy treatment. (**d**) Prognostic significance of SMOC2 expression was more apparent in stage III colon cancer patients. Scale bar: 100 μm.

IHC for SMOC2 was performed on 12 tissue microarrays and in total 591 cases were included for survival analysis. CRCs with H-scores of more than 40 were defined as positive for SMOC2 (Fig. 4a). The clinicopathological relevance of SMOC2 positivity is summarized in Table 1. SMOC2 expression was negatively associated with lymphatic invasion (*P* = 0.002), venous invasion (*P* = 0.002), and tumor stage (*P* < 0.001), while being positively associated with nuclear β-catenin expression (*P* = 0.030). SMOC2 had no correlations with age, gender, location, and tumor differentiation. The molecular pathogenesis of CRCs has been classified into three or four groups by distinct characteristics such as CIMP, MSI, and mutation status of either BRAF or KRAS. However, no significant association was observed between SMOC2 and molecular features (Table 2). In addition, we determined the prognostic impact of SMOC2 with a large number of CRC patients, and demonstrated that CRC patients with SMOC2 positivity has better clinical outcomes in both overall (*P* < 0.001), and recurrence-free survival rates (*P* < 0.001) (Fig. 4b), regardless of adjuvant chemotherapy treatment (Fig. 4c). The prognostic significance of SMOC2 was more apparent in stage III CRC patients than in stage I, II or IV CRC patients (Fig. 4d). Furthermore, multivariate analysis revealed that SMOC2 was an independent prognostic marker (HR = 0.555, *P* = 0.006) along with perineural invasion (HR = 1.890, P < 0.001), tumor stage (HR = 3.029, *P* < 0.001), and adjuvant chemotherapy (HR = 0.281, P < 0.001) (Table 3).

**Table 1:** Association between SMOC2 expression and the clinicopathological characteristics.

Characteristic	Total (%)	SMOC2		*P-*value
		Negative (%)	Positive (%)	
Patients	591 (100)	424 (72)	167 (28)	
Number of death events	170 (100)	142 (84)	28 (16)	
**Age**				
≥ 60	336 (57)	235 (70)	101 (30)	0.270^†^
< 60	255 (43)	189 (74)	66 (26)	
**Gender**				
Female	231 (39)	166 (72)	65 (28)	0.100^†^
Male	360 (61)	258 (72)	102 (28)	
**Location**				
Proximal	158 (27)	114 (72)	44 (28)	0.918^†^
Distal	433 (73)	310 (72)	123 (28)	
**Differentiation**				
WD	44 (7)	30 (68)	14 (32)	0.862^#^
MD	529 (90)	381 (72)	148 (28)	
PD	18 (3)	13 (72)	5 (28)	
**Lymphatic invasion**				
Negative	334 (57)	223 (67)	111 (33)	0.002^†^
Positive	257 (43)	201 (78)	56 (22)	
**Venous invasion**				
Negative	512 (87)	356 (70)	156 (31)	0.002^†^
Positive	89 (13)	68 (86)	11 (14)	
**Perineural invasion**				
Negative	449 (76)	313 (70)	136 (30)	0.055^†^
Positive	142 (24)	111 (78)	22 (22)	
Tumor stage*				
I	92 (16)	43 (47)	49 (53)	< 0.001^#^
II	193 (33)	131 (68)	62 (32)	
III	209 (35)	167 (80)	42 (20)	
IV	97 (16)	83 (86)	14 (14)	
**β-catenin**				
No nuclear stain	222 (38)	171(76)	51 (24)	0.030^†^
Nuclear stain	369 (62)	253 (69)	116 (31)	

*WD* well differentiated, *MD* moderately differentiated, *PD* poorly differentiated.

^†^Fisher's exact test. ^#^ Pearson chi-square test, *AJCC 7th edition.

**Table 2:** Association between SMOC2 expression and molecular characteristics.

Characteristic	Total (%)	SMOC2		*P*-value
		Negative (%)	Positive (%)	
Patients	603 (100)	433 (72)	170 (28)	
CIMP				
Negative & low	563 (93)	400 (71)	163 (29)	0.146^†^
High	40 (7)	33 (83)	7 (17)	
MSI				
Negative & low	550 (91)	396 (72)	154 (28)	0.750^†^
High	53 (9)	37 (70)	16 (30)	

	Total (%)	SMOC2		*P*-value
		Negative (%)	Positive (%)	
Patients	571 (100)	414 (73)	157 (27)	
KRAS				
Wt	415 (73)	303 (73)	112 (27)	0.675^†^
Mt	156 (27)	111 (71)	45 (29)	

	Total (%)	SMOC2		*P*-value
		Negative (%)	Positive (%)	
Patients	598 (100)	431 (72)	167 (28)	
BRAF				
Wt	568 (95)	410 (72)	158 (28)	0.835^†^
Mt	30 (5)	21 (70)	9 (30)	

*CIMP* CpG island methylator phenotype, *MSI* microsatellite instability, *Wt* wild type, *Mt* mutation.

^†^Fisher's exact test. ^#^Pearson chi-square test.

**Table 3:** The results of univariate and multivariate analysis for overall survival rate in colorectal cancers.

Variables		Univariate analysis			Multivariate analysis		
		HR	95% CI	*P*-value	HR	95% CI	*P*-value^a^
Age	> 60/ < 60	1.309	0.962–1.781	0.086	1.394	0.992–1.958	0.056
Gender	Female/male	0.772	0.563–1.061	0.111			
Site	Distal/proximal	0.755	0.547–1.043	0.089	0.726	0.511–1.031	0.074
Differentiation	Poor/moderate/well	2.389	1.457–3.919	0.001	1.318	0.763–2.276	0.323
Lymphatic invasion	Positive/negative	2.2	1.619–2.989	< 0.001	0.988	0.707–1.381	0.944
Venous invasion	Positive/negative	3.122	2.228–4.375	< 0.001	1.439	0.997–2.078	0.052
Perineural invasion	Positive/negative	3.432	2.733–4.309	< 0.001	1.890	1.336–2.676	< 0.001
Nuclear β-catenin	Positive/negative	0.964	0.708–1.314	0.817			
KRAS mutation	Present/absent	1.025	0.726–1.447	0.887			
BRAF mutation	Present/absent	1.333	0.703–2.527	0.378			
CIMP	High/low or negative	1.84	1.114–3.038	0.017	1.067	0.608–1.872	0.822
MSI	Unstable/Stable	0.84	0.467–1.510	0.559			
Stage	IV/III/II/I	2.972	2.438–3.623	0.000	3.029	2.423–3.787	< 0.001
Adjuvant CTx or CCRT	Yes/No	0.853	0.621–1.172	0.326	0.281	0.200–0.395	< 0.001
SMOC2	Positive/negative	0.46	0.307–0.0690	< 0.001	0.555	0.364–0.848	0.006

*HR* Hazard ratio, *CI* confidence interval, *CIMP* CpG island methylator phenotype, *MSI* microsatellite instability, *CTx* chemotherapy, *CCRT* concurrent chemoradiation therapy.

^a^Cox proportional hazard model.

### Effects of SMOC2 expression on the growth and migration of CRC cells

**Figure 5:**
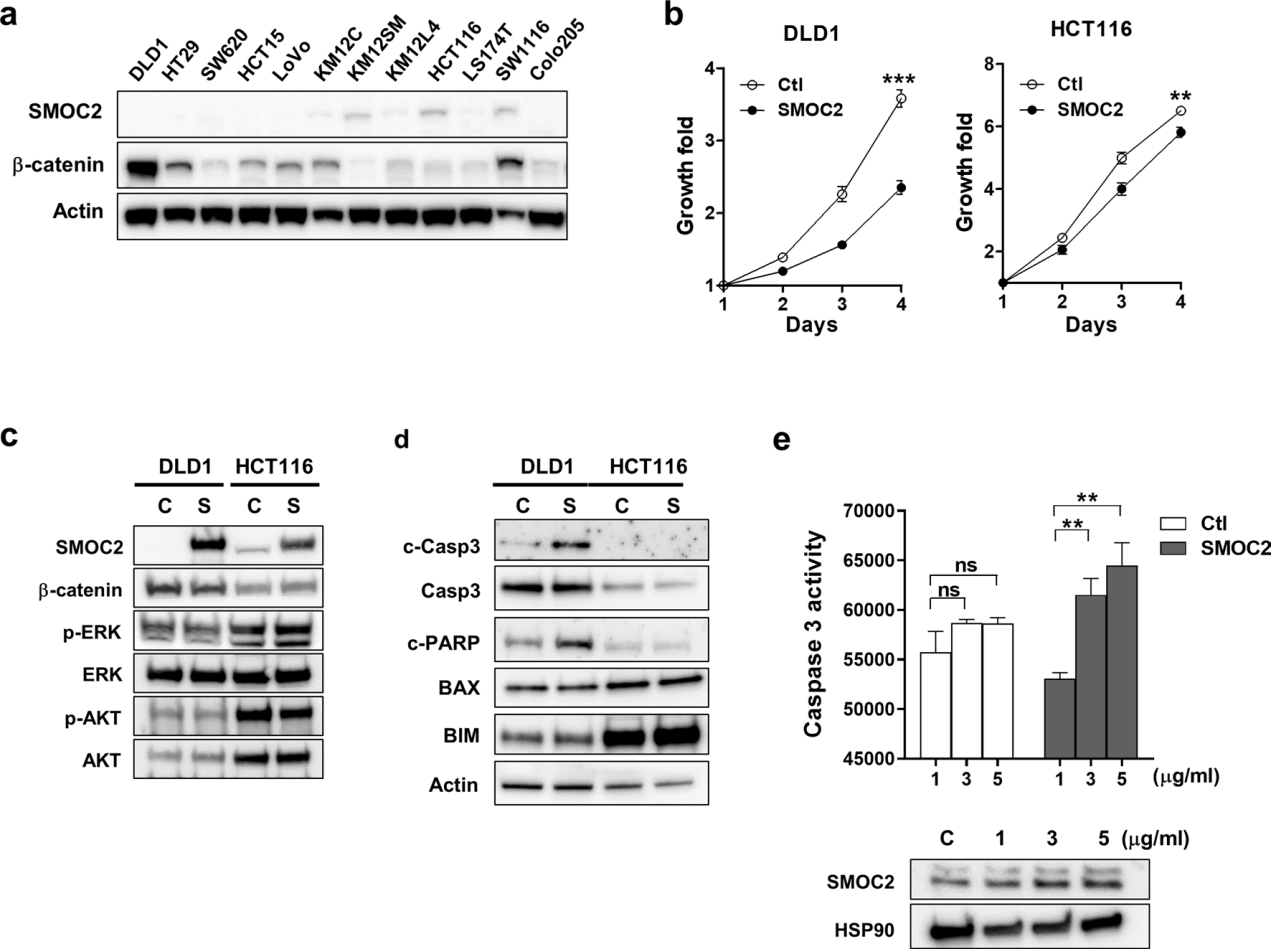
The suppressive effects of SMOC2 overexpression on colorectal cancer (CRC) cell growth. (**a**) The protein levels of SMOC2 in 12 human CRC cell lines were assessed by immunoblot assays. (**b**) CRC cell lines (DLD1 and HCT116) with low level of SMOC2 were transfected with a control or a SMOC2-expressing plasmid. Cell growth was determined using the Cell counting Kit-8 at the indicated times. (**c**) 24 h after transfection with a control or a SMOC2-expressing plasmid in DLD1 and HCT116, immunoblot assay was performed using the antibodies indicated in the figure. (**d**) Immunoblot assay for apoptosis-related proteins was performed in DLD1 and HCT116 after transfection with a control or a SMOC2-expressing plasmid. (**e**) Caspase-3 activity was measured after transfecting DLD1 cells with varying concentrations of control or SMOC2-expressing plasmid (1, 3, 5 µg/mL). Ctl, control; S, SMOC2. Data are presented as the mean ± SD. **P < 0.01, ***P < 0.001.

**Figure 6:**
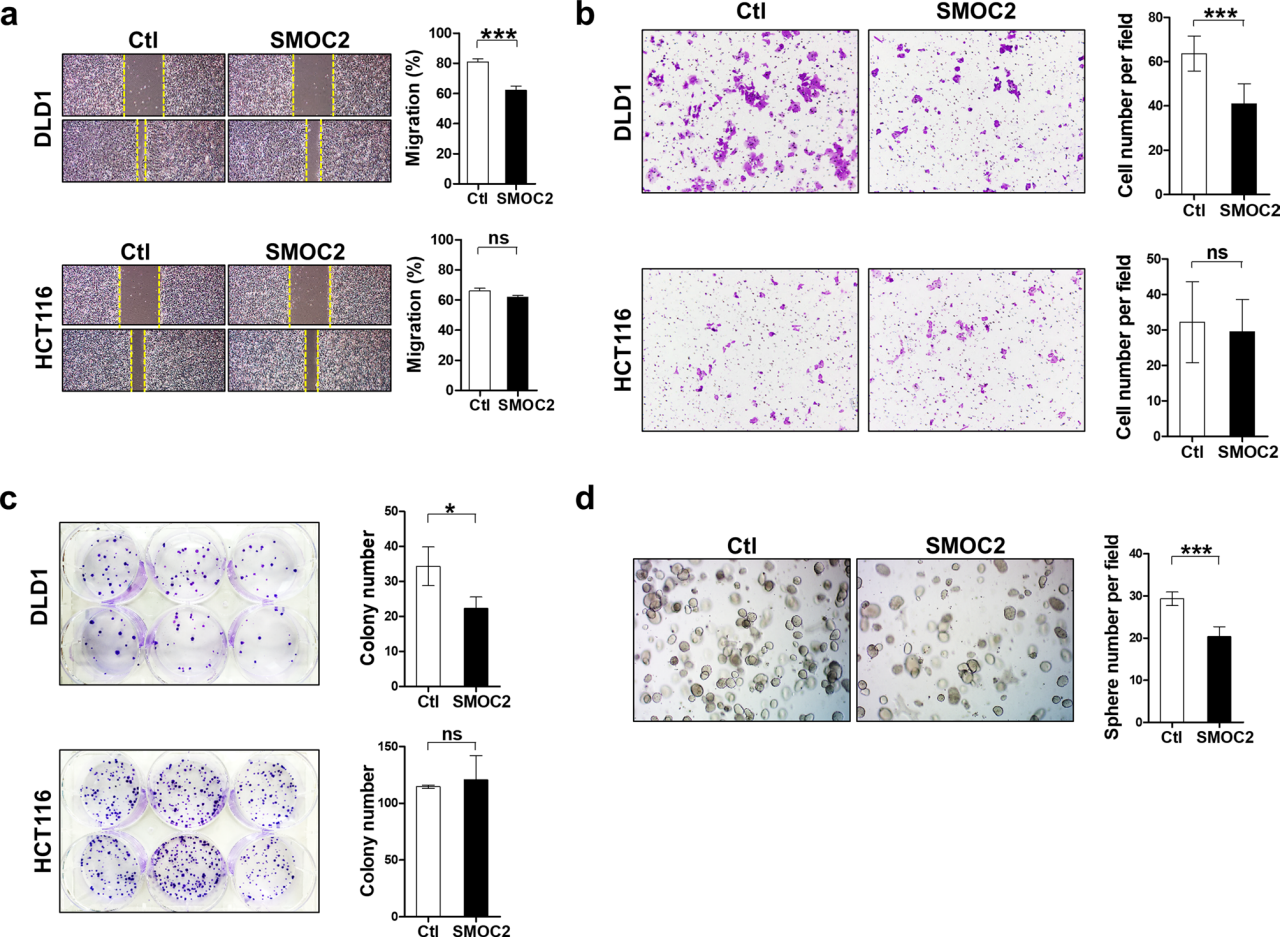
The inhibitory effects of SMOC2 on colorectal cancer cell migration, colony forming and sphere forming activities. (**a**, **b**) The effect of SMOC2 expression on migration activity of DLD1 and HCT116 cells was evaluated by wound healing and transwell migration assays. Cellular migration was photographed at 0 and 48 h. (**c**) Colony forming assay was performed by counting the number of colonies from control or SMOC2- expressing DLD1 or HCT116 cells. (**d**) Sphere forming assay was performed by counting the number of spheres growing from control or SMOC2-expressing DLD1 cells on a three-dimensional matrix. Data are presented as the mean ± SD. Ctl, control. *P < 0.05, ***P < 0.001.

To explore the functional roles of SMOC2 in CRCs, we screened 12 CRC cell lines and found that SMOC2 protein levels are undetectable or very low in seven cell lines, while three cell lines, includingKM12SM, HCT116 and SW1116, showed significant SMOC2 expression (Fig. 5a). The positive association of SMOC2 with nuclear β-catenin expression, found in our IHC analysis, was not observed in immunoblot analysis of CRC cell lines (Fig. 5a). To determine the influence of SMOC2 on CRC growth, we induced SMOC2 over-expression in DLD1 and HCT116 cell lines. DLD1 and HCT116 cell lines were selected since they express low level of SMOC2 and have excellent transfection efficiency. We observed a significantly attenuated proliferation profile in cancer cells that were transfected with SMOC2, compared to those transfected with a control plasmid (Fig. 5b). As cancer cell survival often involves AKT or MAPK signaling pathways, we examined the activation of AKT and ERK proteins. However, no significant alteration was observed in neither phospho-ERK nor phospho-AKT levels (Fig. 5c). However, cleaved caspase-3 and PARP expression increased by SMOC2 over-expression in DLD1, indicating that SMOC2-induced growth suppression may involve activation of apoptotic pathways (Fig. 5d). This finding was confirmed in DLD1 by enhanced caspase-3 activity along with increasing concentrations of SMOC2-expressing DNA (Fig. 5e). Decreased migratory activity was demonstrated in the wound healing and transwell migration assays upon SMOC2 transfection in DLD1 (Fig. 6a,b). Furthermore, SMOC2 overexpression in DLD1 showed a substantial decrease in colony and sphere forming abilities (Fig. 6c,d). Unlike DLD1, SMOC2 expression in HCT116 showed no significant difference in the wound healing, migration, or colony formation assays. To examine whether the tumor suppressive functions of SMOC2 are mediated through the secreted SMOC2 protein in an extracellular space, we treated DLD1 with recombinant human SMOC2 protein. However, addition of recombinant SMOC2 protein into the culture media had no significant impact on the growth of DLD1 (Fig. S1).

## Discussion

In this study, we thoroughly investigated the expression profile of SMOC2 in various colorectal precancerous lesions as well as in a large cohort of human CRCs, and demonstrated that SMOC2 is an independent prognostic marker associated with improved clinical outcomes. In agreement with its prognostic effect, we discovered that SMOC2 overexpression in colon cancer cells resulted in significantly attenuated proliferation, migration, and colony forming abilities, suggesting a tumor suppressive role of SMOC2 in cancer progression. However, considering that SMOC2 expression is substantially higher in precancerous colorectal lesions such as TSAs and TAs, it is less likely that SMOC2 exerts its tumor suppressive function at the early stage of tumor development. This observation is contrary to previous studies that also report on the oncogenic properties of SMOC2. For example, the induction of SMOC2 was required to increase cell growth, motility, and metastasis in L1-expressing CRC cells^12^, and SMOC2 suppression in breast cancer cells decreased the ability of the Ran mutant to stimulate anchorage-independent growth^24^. In addition, SMOC2 knockdown in lung adenocarcinoma cells led to reduced clonal growth and metastatic seeding^14^. This discrepancy may be due to the difference in either the cellular context or cell lines used in the in vitro study. Indeed, the opposite functional effects of SMOC2 have been reported in hepatocellular carcinomas (HCCs); Huang et al. reported that SMOC2 over-expression in HepG2 cells inhibited cell proliferation, migration, and invasion^25^, whereas Su et al. demonstrated that SMOC2 promoted cell proliferation through the cell cycle progression in other HCC cell lines, including MHCC-97H and Huh7 cells^13^. Therefore, further studies are required to reveal the functional significance of SMOC2 expression in each cancer type and, in particular, to confirm our results of tumor suppressive roles in CRCs.

In the mouse small intestine, SMOC2 was proven to be specifically expressed in the stem cells of the crypt. Here, in the human small and large intestines we observed the same localization of SMOC2-positive cells, confined to the crypt bases, suggesting that SMOC2 is likely to be an ISC marker of human intestines. In HPs and SSAs, SMOC2 expression was still restricted to the crypt bases right above muscularis mucosa, whereas in TSAs and TAs SMOC2 expression was not confined to the crypt bottom any more. Interestingly, ectopic crypt foci (ECF), a distinct histologic feature of TSAs, tend to exhibit strong SMOC2 expression, suggesting ECF to be spots where groups of cell populations with stem cell phenotypes exist. TAs showed strong SMOC2 expression in a patchy or diffuse manner. This difference in the SMOC2 expression pattern between HPs/SSAs and TSAs/TAs might exhibit distinctive characteristics in regards to stem cell hierarchy among precancerous lesions; the loss of restriction of ISC marker expression may represent a more advanced stage of TSAs/TAs than HPs/SSAs in tumor development.

Interestingly, it was not uncommon to find the nuclear staining of SMOC2 in TSAs and TAs. To examine whether SMOC2 protein can be translocated to the nucleus when its expression is highly induced, we transfected DLD1 cells with SMOC2-expressing plasmid and evaluated SMOC2 levels in the nuclear and cytoplasmic fractions. However, SMOC2 protein was only detected in the cytoplasm (Fig. S2). Based on this finding, only cytoplasmic staining was considered positive when interpreting the IHC results.

As SMOC2 is one of the enriched genes in stem cells of the normal crypts, we examined whether there is any correlation between SMOC2 and other established ISC markers including LGR5, ASCL2, EPHB2, and OLFM4. However, only a weak positive correlation between SMOC2 and OLFM4 was observed; this is consistent with the previous study in which LGR5 expression levels, one of the most important ISC markers, had no association with other ISC signature genes in CRCs^22^. These results suggest that the stem cell hierarchy in the normal crypts represented by the enrichment of ISC signature genes is likely to be lost during colorectal carcinogenesis. Furthermore, we did not find any association between SMOC2 and well-known candidate CSC makers, such as CD24, CD44, CD133, and CD166, indicating that SMOC2-positive cells are less likely to overlap with the candidate CSC population. Considering that SMOC2 is an ISC marker, there is a possibility that SMOC2 might act as a CSC marker in CRCs. However, technically, it would not be easy to isolate and characterize SMOC2-positive cells for a CSC assay as SMOC2 is a cytosolic and secreted glycoprotein and not a membranous protein.

Many intestinal stem cell markers such as LGR5, ASCL2, and EPHB2 are the direct Wnt target genes, and we previously showed positive associations of LGR5 and EPHB2 with nuclear β-catenin in CRCs^21,22^. Although SMOC2 is not one of Wnt target genes, in this study we observed a significant association between SMOC2 and nuclear β-catenin. SMOC2 expression may be indirectly affected by increased Wnt signaling activity. Recently, it has been reported that SMOC-2 directly interacts with WNT receptors, enhances ligand-receptor interaction, and finally activates the WNT/β-catenin pathway in endometrial cancer^15^. Thus, it is also possible to hypothesize that SMOC2 expression may contribute to increased Wnt signaling activity in CRCs.

By directly comparing the H-scores of SMOC2 between adenoma and carcinoma areas that co-exist in the same tumor, we found that high levels of SMOC2 expression was persistent during the adenoma to carcinoma transition. It was extremely rare to see the case where SMOC2 expression is higher in carcinoma areas than in pre-existing adenoma areas. Furthermore, it is noteworthy to highlight that we found SMOC2 expression to be significantly reduced when cancer cells invaded into deeper layers and did not further decline during lymph node metastasis. This is consistent with the result of our migration assay, in which induced-SMOC2 over-expression led to decreased migratory activity of colon cancer cells, even though it was only observed in DLD1 cells among the two cell lines examined. When examining the down-stream signaling molecules, SMOC2 over-expression showed no alterations in β-catenin, phospho-ERK, and phospho-AKT levels, indicating Wnt, MAPK, or AKT pathways are not involved in SMOC2-induced growth and migration suppression. However, SMOC2 increased the levels of cleaved caspase-3 and PARP, suggesting pro-apoptotic roles of SMOC2; this finding has been confirmed by a caspase-3 assay where caspase-3 activity was shown to increase with rising concentrations of transfected SMOC2-expressing DNA. As BAX and BIM expression remained the same upon SMOC2 overexpression, it is less likely that SMOC2-induced apoptosis is mediated by intrinsic apoptosis pathways of the Bcl-2 family.

To further investigate the underlying mechanism of SMOC2-induced tumor suppressive effects in colon cancer cells, we performed cDNA microarray analysis using SMOC2-expressing stable cell lines. Among the genes that are down-regulated by SMOC2 expression, we focused on two genes that are involved in cancer growth and migration, *DDX46* and *CEACAM5*. Subsequent real-time PCR analysis was only able to confirm downregulation of *CEACAM5* in SMOC2-expressing cell lines (Fig. S3). *CEACAM5* codes for CEA, which is a well-known intercellular adhesion molecule that is known to be over-expressed in a majority of carcinomas, and plays important roles in cancer invasion and metastasis^26^. Considering the established oncogenic roles of CEACAM5, it is reasonable to speculate that SMOC2-induced CEACAM5 down-regulation may contribute to a tumor suppressive role of SMOC2 in CRC progression.

Several limitations of our study should be acknowledged. As a retrospective study, the regimens of adjuvant chemotherapy delivered to the patients widely varied as described earlier and some patients even received radiation therapy. This may have acted as a confounding factor that affected the survival rate even though we observed a significant prognostic impact of SMOC2 in CRC patients. To our knowledge, this is the first study investigating the prognostic value of SMOC2 in a large cohort of CRC patients, therefore it is necessary to validate our findings in an independent CRC cohort. In the in vitro functional studies, we did not reach the conclusive results concerning the effects of SMOC2 on CRC growth. DLD1 cells showed substantially declined capabilities both in growth and migration upon SMOC2 overexpression; however, consistent results were not observed in other cell lines such as HCT116, HT29, and SW620 (Figs. 6 and S4). This may suggest that SMOC2 does not play a dominant role in regulation of cancer progression. Further meticulous studies are required to clarify the functional roles of SMOC2 in CRC progression. In addition, the cell lines used were not tested for mycoplasma, which may have influenced the results of functional experiments.

In summary, high levels of SMOC2 expression was observed in TSAs and TAs among precancerous lesions. Up-regulated SMOC2 expression remained persistent during adenoma-carcinoma progression, however, it significantly decreased while invading the deeper layers. Notably, SMOC2 was an independent prognostic marker for improved clinical outcomes in CRCs. In vitro assays showed that SMOC2 over-expression suppressed proliferation and migration, as well as colony and sphere forming abilities in some colon cancer cell lines. Taken together, our results suggest that SMOC2 may act as a tumor suppressor in CRC progression.

## Supplementary information

Supplementary Information.
