# Supplementary material for: SMOC2, an intestinal stem cell marker, is an independent prognostic marker associated with better survival in colorectal cancers
Source: Sci Rep. 2020 Sep 3;10:14591. doi: 10.1038/s41598-020-71643-1 (PMC7471277; doi:10.1038/s41598-020-71643-1)

SMOC2, an intestinal stem cell marker, is an independent prognostic marker associated with better survival in colorectal cancers

(Running head: Prognostic value of SMOC2 in colorectal cancers)

Bo Gun Jang[1, #], Hye Sung Kim[1, #], Jeong Mo Bae[3, 4], Woo Ho Kim[3], Heung Up Kim[2], Gyeong Hoon Kang[3, 4]

[1]Department of Pathology, Jeju National University School of Medicine, Jeju, Korea

[2]Department of Internal Medicine, Jeju National University School of Medicine, Jeju, Korea;

[3]Department of Pathology, Seoul National University College of Medicine, Seoul, Korea

[4]Laboratory of Epigenetics, Cancer Research Institute, Seoul National University College of Medicine, Seoul, Korea

[#]These authors contributed equally to this work.

Corresponding Author

Gyeong Hoon Kang

Department of Pathology, Seoul National University College of Medicine

103 Daehak-ro, Jongno-gu, Seoul 110-799, Korea

ghkang@snu.ac.kr

Heung Up Kim

Department of Internal Medicine, Jeju National University School of Medicine

Aran 13gil 15, Jeju 690-767, Korea

kimhup@hanmail.net

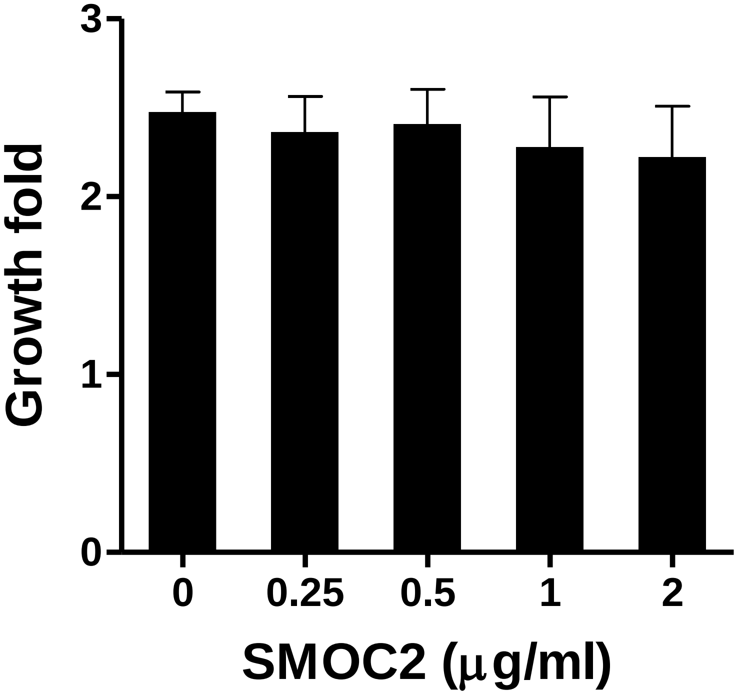

Supplementary Fig. 1 Effect of SMOC2 protein treatment on the growth of colon cancer cells. Recombinant human SMOC2 protein was added into the culture media showed no difference in the proliferation of DLD1 cells.

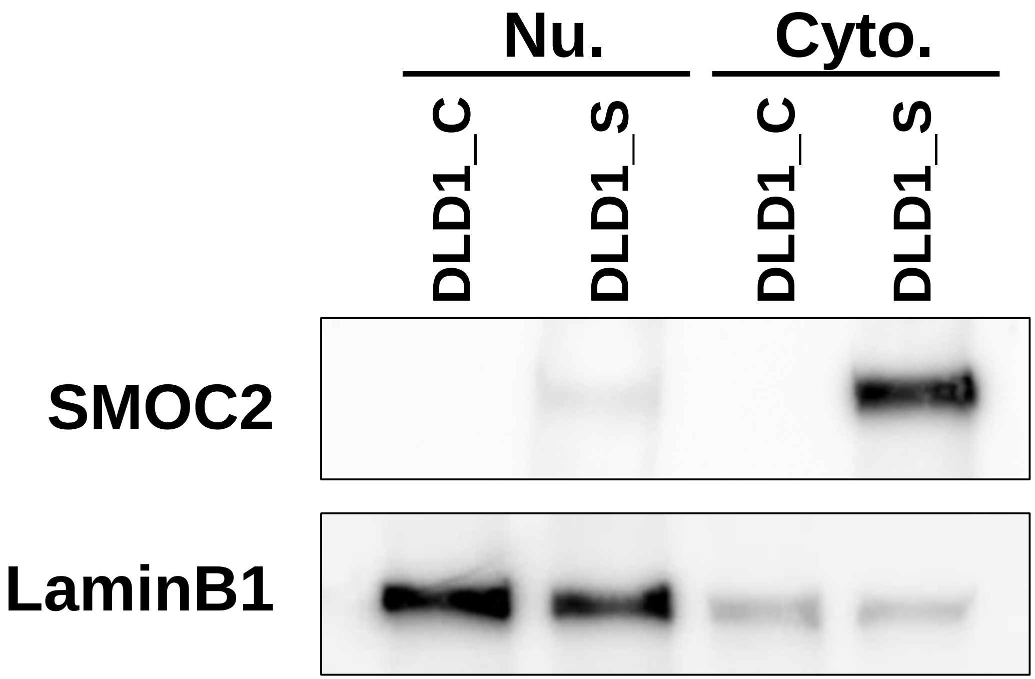

Supplementary Fig. 2 The protein levels of SMOC2 in the nuclear and cytoplasmic fractions of control vector- (DLD1_C) or SMOC2-expressing DLD1 cells (DLD1_S). Nu, Nuclear; Cyto., Cytoplasmic.

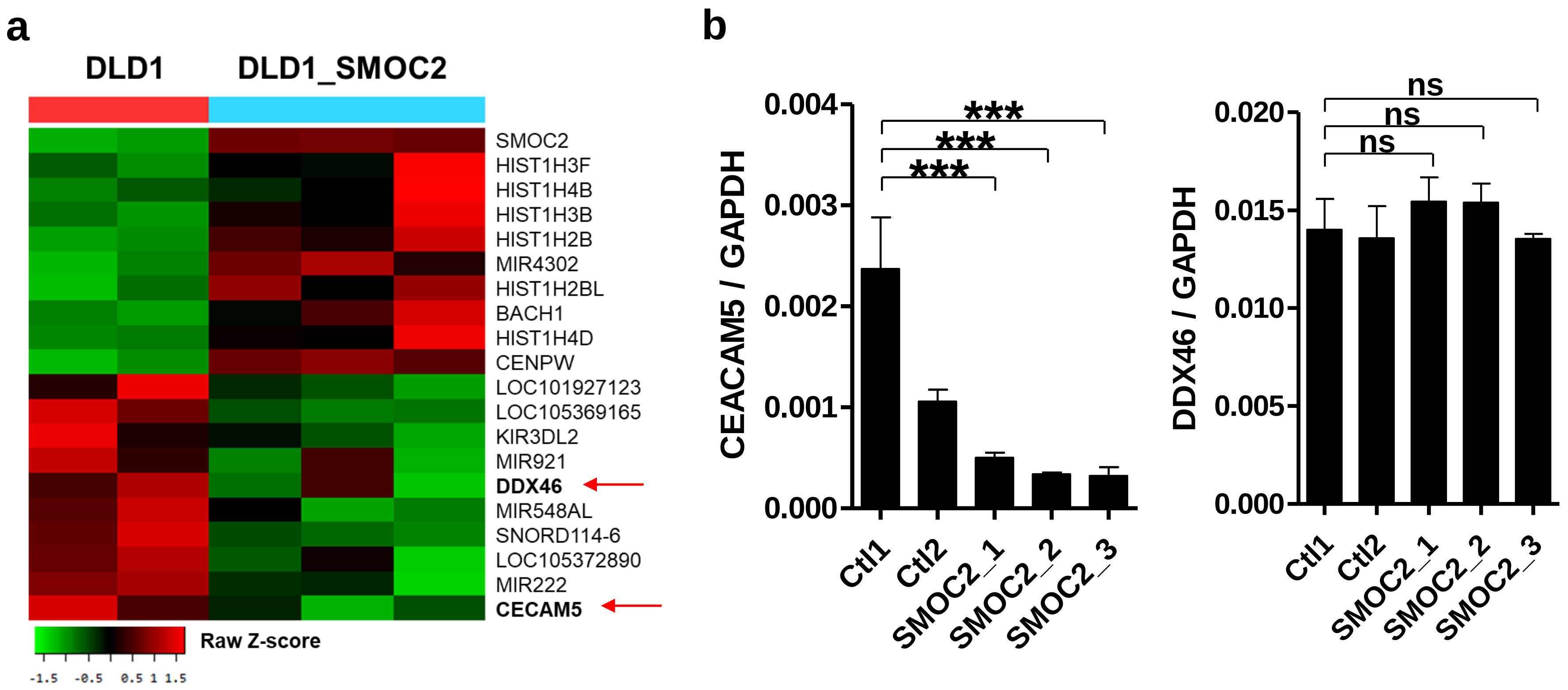

Supplementary Fig. 3 To identify the genes that are altered by SMOC2 overexpression, cDNA microarray was performed with DLD1 cells stably transfected with control (Ctl1 and Ctl2) or SMOC2-plasmid vector (SMOC2_1, SMOC2_2, and SMOC2_3). (a) Among top ten genes downregulated by SMOC2 overexpression, two genes, DDX46 and CEACAM5 were identified as cancer-related genes. (b) Only CEACAM5 was confirmed to decrease upon SMOC2 overexpression by real time-PCR.

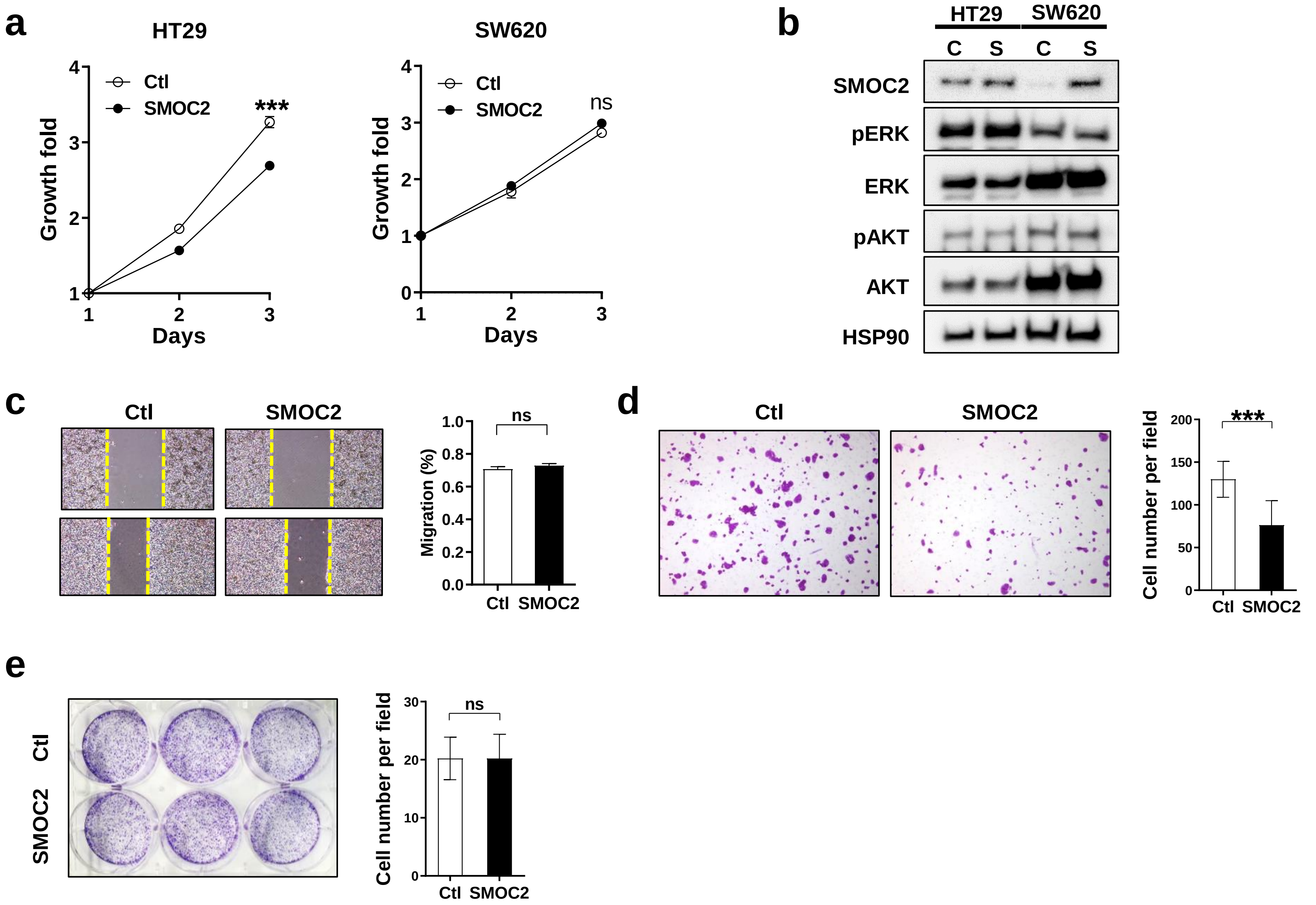

Supplementary Fig. 4 The effects of SMOC2 overexpression in HT29 and SW620 cells. (a) After transfection with a control or a SMOC2 expressing-plasmid, cell growth was measured at the indicated times. (b) 24 hours after transfection, immunoblot assay was performed with the antibodies indicated in the figure. C, control; S, SMOC2. (c,d) The effect of SMOC2 expression on migration activity of HT29 cells was evaluated by Wound healing and Transwell migration assays. Cellular migration was photographed at 0 and 48 h. (e) Colony forming activity was determined by counting the number of colonies from control or SMOC2 expressing-HT29 cells. Data are presented as the mean ± SD. Ctl, control; ns, not significant; $^{***}P < 0.001$.

Supplementary Fig. 5 Original images of Western blot.

Fig. 5a SMOC2

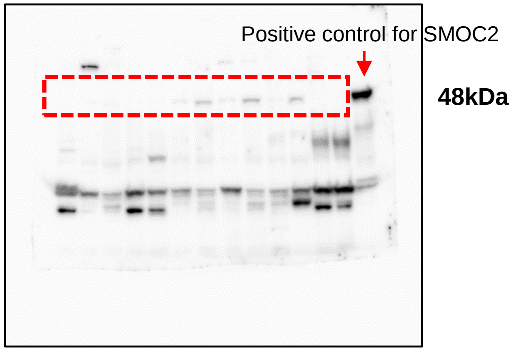

Fig. 5a β-catenin, Actin

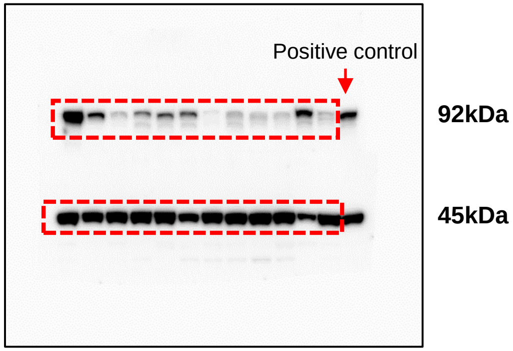

Fig. 5c SMOC2

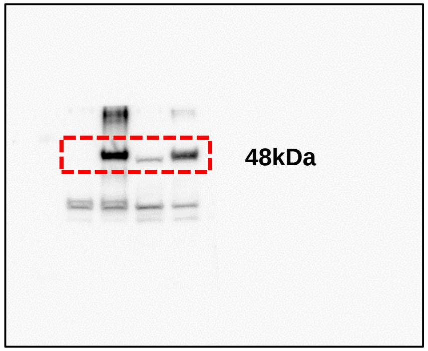

Fig. 5c β-catenin

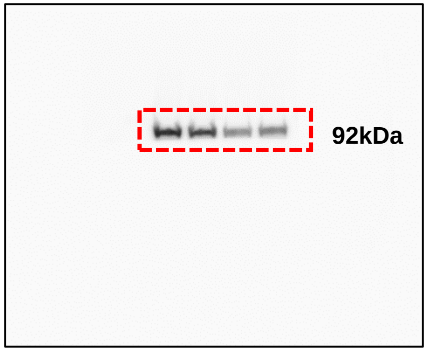

Fig. 5c p-ERK

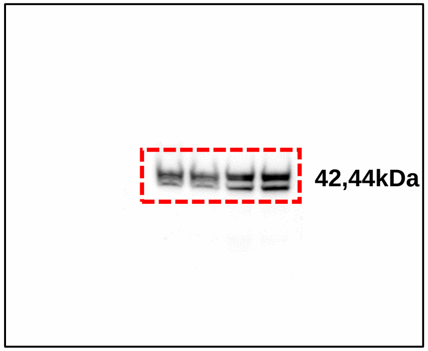

Fig. 5c AKT, ERK

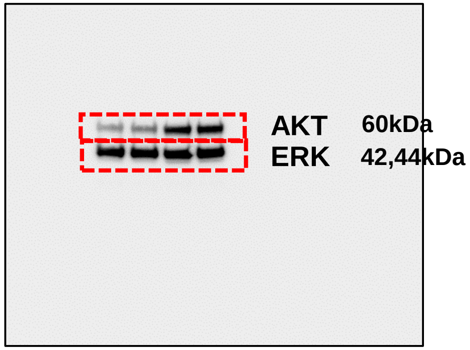

Fig. 5c p-AKT

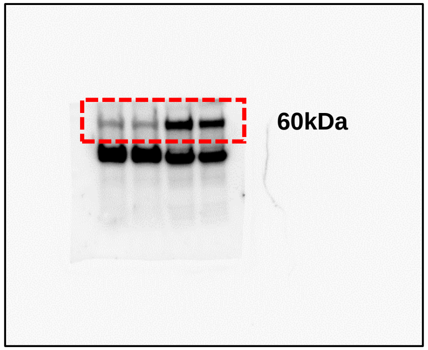

Fig. 5d cleaved-Caspase 3

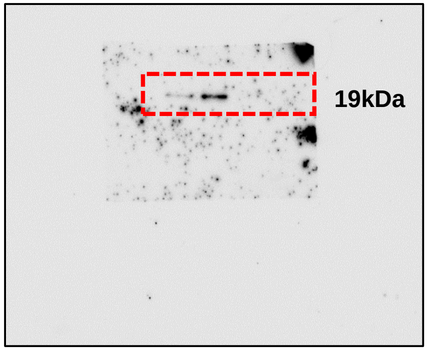

Fig. 5d Caspase 3

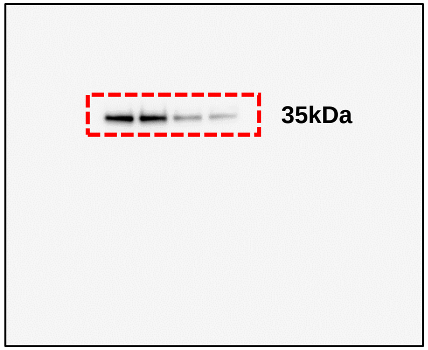

Supplementary Fig. 5 Original images of Western blot.

Fig. 5d cleaved PARP

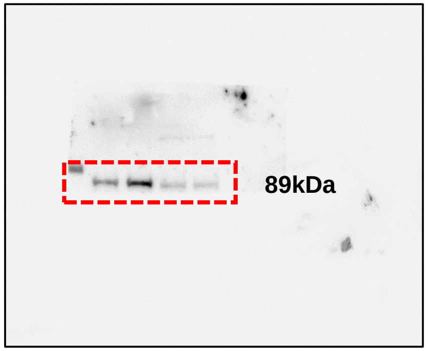

Fig. 5d BAX

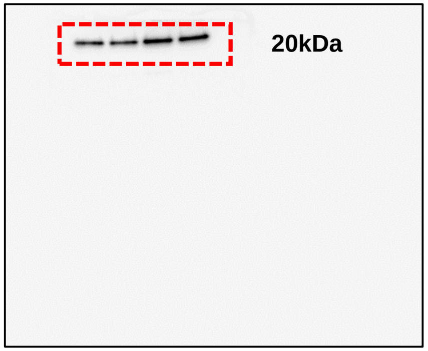

Fig. 5d BIM

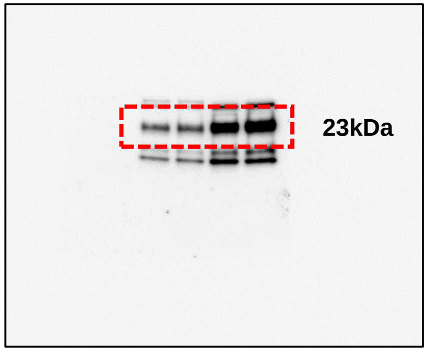

Fig. 5d Actin

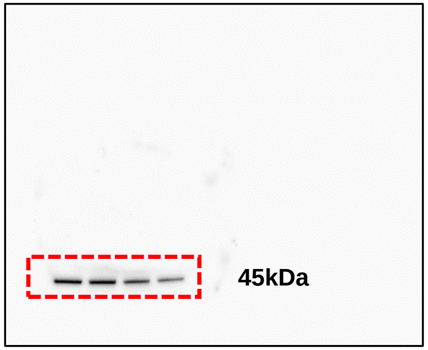

Fig. 5e SMOC2

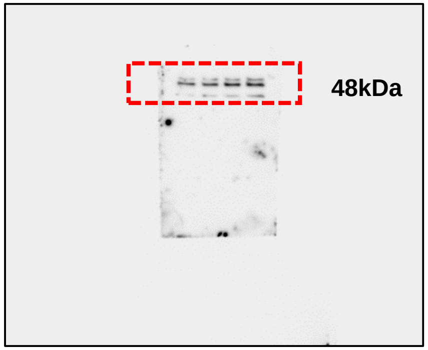

Fig. 5e HSP90

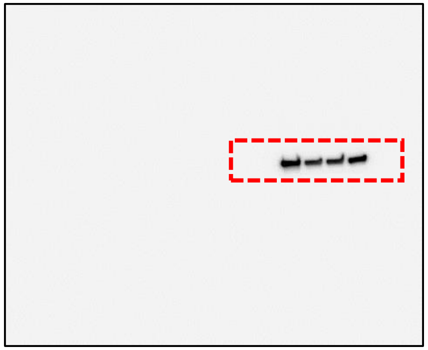

Supplementary Fig. 2 SMOC2

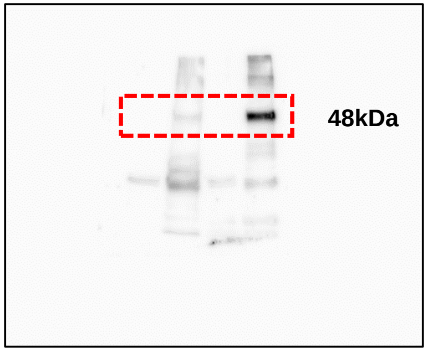

Supplementary Fig. 2 LaminB1

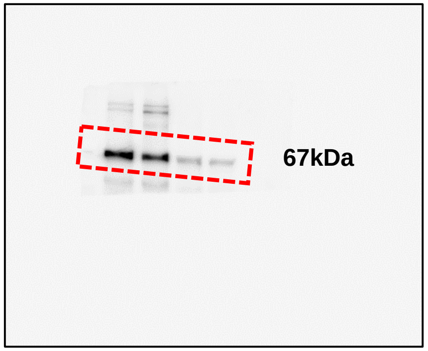

Supplementary Fig. 4 SMOC2

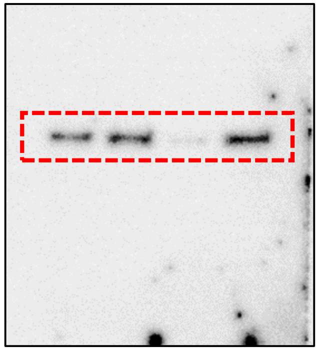

Supplementary Fig. 5 Original images of Western blot.

Supplementary Fig. 4 AKT, ERK

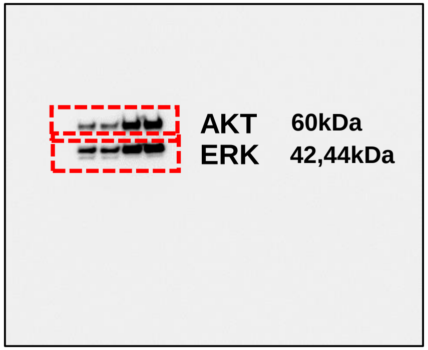

Supplementary Fig. 4 pERK

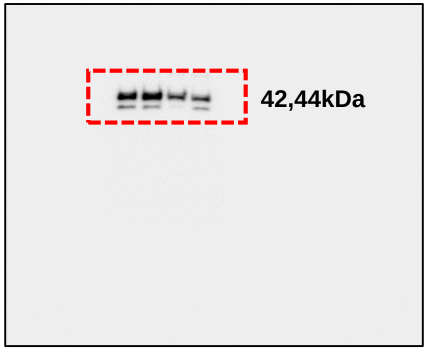

Supplementary Fig. 4 pAKT

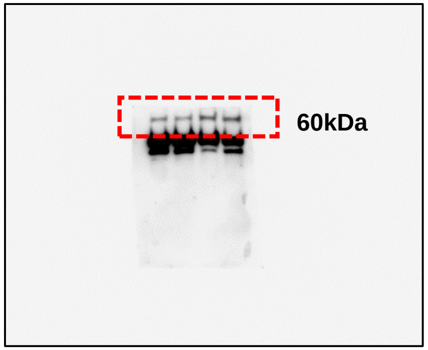

Supplementary Fig. 4 HSP90

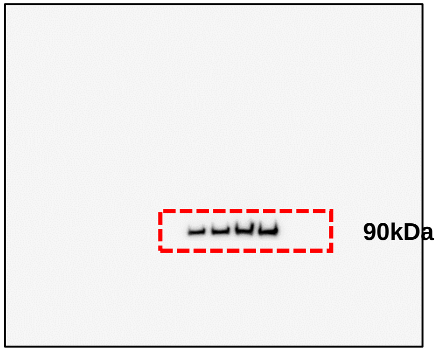

Supplement: Supplementary file 1 — Supplementary Information. [file 41598_2020_71643_MOESM1_ESM.pdf]
